# Ultrasonically Enhanced ZD2767P–Carboxypeptidase G2 Deactivates Cisplatin-Resistant Human Lung Cancer Cells

**DOI:** 10.1155/2022/9191233

**Published:** 2022-11-04

**Authors:** Qianfen Liu, Xinya Li, Yuanyuan Luo, Houmei Wang, Ying Zhang, Tinghe Yu

**Affiliations:** ^1^Department of Obstetrics and Gynecology, Chongqing Health Center for Women and Children (Women and Children's Hospital, Chongqing Medical University), Chongqing, China; ^2^The Second Affiliated Hospital, Chongqing Medical University, Chongqing, China

## Abstract

The prodrug–enzyme regimen ZD2767P+CPG2 is limited by low efficacy. Here, ultrasound was used to modulate ZD2767P+CPG2 (i.e., ZD2767P+CPG2+US) against cisplatin-resistant human lung cancer cells. A549 and A549/DDP (resistant subline) cells received ZD2767P+CPG2 or ZD2767P+CPG2+US. Either ZD2767P+CPG2 or ZD2767P+CPG2+US led to cell death and apoptosis, and ZD2767P+CPG2+US produced stronger effects; comet assays revealed that these two means directly caused DNA double-strand break. Z-VAD-fmk and/or ferrostatin-1 increased the cell survival percentage, and Z-VAD-fmk decreased the apoptosis percentage. The level of transferrin was increased in treated cells, but those of ferroportin and glutathione peroxidase 4 (GPX4) were reduced, with higher intracellular levels of reactive oxygen species and of iron. Intracellular pharmacokinetics of ZD2767D (activated drug) indicated that the peak level, area under the drug level vs. time curve, and mean residence time in ZD2767P+CPG2+US were higher than those in ZD2767P+CPG2. Both ZD2767P+CPG2 and ZD2767P+CPG2+US were effective on xenograft tumors in nude mice; inhibitory rates were 39.7% and 63.5% in A549 tumors and 50.0% and 70.1% in A549/DDP tumors, respectively. A higher apoptosis level and a lower GPX4 level were noted in tumors receiving treatments. No severe adverse events were observed. These data demonstrated that ZD2767P+CPG2+US deactivated cancer cells via apoptosis and ferroptosis pathways, being a candidate therapy for cisplatin-resistant lung cancer.

## 1. Introduction

Cisplatin (DDP) is the first-line chemotherapeutic agent for non-small-cell lung cancer (NSCLC) that accounts for 85% of lung cancer cases. Unfortunately, the development of resistance progressively decreases clinical responses and eventually leads to treatment failure [1, 2]. Cisplatin induces apoptosis to realize cytotoxicity, and apoptosis insufficiency can lead to resistance [2]. Therefore, offsetting the apoptosis deficiency with other cell death modes is a candidate modality to defeat resistance, improving the therapeutic outcome.

As a prodrug–enzyme modality, ZD2767P (4-[N,N-bis(2-iodoethyl)amino] phenoxycarbonyl L-glutamic acid) is converted to ZD2767D (4-[N,N-bis(2-iodoethyl)amino] phenol) by carboxypeptidase G2 (CPG2); ZD2767D attacks DNA to induce apoptosis [3, 4]. The release of ZD2767D can be restricted within cancer tissues via adjusting the distribution field of CPG2 with delivery techniques (e.g., antibody directing and precision delivery), thereby realizing a targeted treatment [5, 6]. Preliminary clinical trials have demonstrated the efficacy of ZD2767P+CPG2. However, extratumoral activation of ZD2767P and the leakage of ZD2767D from tumors back into circulation lead to toxicities; ZD2767D is with a short half-life (about 2 min), resulting in low anticancer efficacy; immunogenicity of CPG2 limits repeated cycles [7–9]. These bottlenecks have hindered development of the ZD2767P+CPG2 therapy.

Ultrasound (US) can permeabilize the vessels and cell membranes, which facilitates the influx of drugs into cancer cells to enhance the action of those drugs (i.e., sonochemotherapy) [10, 11]. Ultrasound can biphasically modulate the activity of CPG2, thereby being a means to regulate the enzymatic reaction to adjust the output rate of the product [12]. Ultrasound was used to enhance ZD2767P+CPG2 (i.e., ZD2767P+CPG2+US) against cisplatin-resistant human ovarian cancer cells in our previous study. ZD2767P+CPG2+US produced higher in vitro and in vivo efficacy in comparison with ZD2767P+CPG2, with good tolerability in mice bearing xenograft tumors [13]. These data have manifested that ZD2767P+CPG2+US is a promising therapeutic modality for resistant ovarian cancer.

Mechanisms of cisplatin resistance vary between cancer types, e.g., nucleotide excision repair (NER) playing the leading part in DNA repair in NSCLC but homologous recombination (HR) being the major pathway in ovarian cancer; consequently, a means that can overcome resistance in a specific cancer type is unnecessarily effective in another cancer type [14–16]. Therefore, whether ZD2767P+CPG2+US can treat cisplatin-resistant NSCLC was explored in this study. Preliminary data indicated that ZD2767P+CPG2+US deactivated resistant cancer cells via apoptosis and ferroptosis pathways.

## 2. Materials and Methods

### 2.1. Cells

NSCLC cell lines A549 and the A549/DDP (identified by STR; Cell Bank, Type Culture Collect., Chin. Acad. Sci., Shanghai, China) were cultured in RPMI 1640 medium (GIBCO, Beijing, China) supplemented with 10% fetal bovine serum (Biol. Ind., Kibbutz Beit Haemek, Israel), at 37°C and 5% CO_2_. A549/DDP was a resistant subline that can grow at 2.0 *μ*g/mL of cisplatin, and cells were transferred to cisplatin-free medium for 5 days to remove residual drugs before experiments [17, 18]. Cells were digested with trypsin, suspended in serum-free medium (1.0 × 10^6^ cells/mL), and then exposed to drugs and insonation.

### 2.2. Insonation

The cell suspension (1.0 mL) was insonated (1.0 MHz, 10 W/cm^2^ for 20 s in continuous waves) as described previously using a device designed and calibrated by Chongqing Haifu Med. Technol. Co. Ltd. (Chongqing, China), with prechilled (4°C) water as the coupling medium [12, 13]. Insonation induced no cytotoxicity and no temperature rise (<37°C) in the sonicated medium (Figure S1) [12]. This device was also adopted for in vivo therapies.

### 2.3. In Vitro Therapies

Five experimental groups were set: cells received ZD2767P, ZD2767P and insonation, ZD2767P combined with CPG2, and the combination of ZD2767P and CPG2 followed by insonation, in groups ZD2767P, ZD2767P+US, ZD2767P+CPG2, and ZD2767P+CPG2+US, respectively. Control cells were subjected to isoconcentration of dimethylsulfoxide (DMSO) (<0.1%) and sham insonation, because the stock solution of ZD2767P and inhibitors was prepared with DMSO. Insonation was performed immediately after adding drugs, drugs were washed away after 1 h, and then cells were cultured in complete medium. Therefore, both the concentration and “concentration × time” tallied with human pharmacokinetics (PK) [19]. Cell viability was determined after 72 h using the WST-8 assay (Dojindo Lab., Kumamoto, Japan), and the percentage of survival cells and IC_50_ (half maximal inhibitory concentration) were calculated. The interaction between ZD2767P+CPG2 and ultrasound was assessed with the combination index (CI) that was calculated using percentages of dead cells (1 – survival percentage). A CI of >1.15 indicated synergism [20].

ZD2767P (WuXi AppTec, Shanghai, China) was 2.5–160 *μ*M in cytotoxicity trials and was 10 *μ*M in other trials. CPG2 (Pseudomonas sp. RS-16 origin; Chongqing Kerun Biomed. Pharm., Chongqing, China) was 1.2 U/mL, having no cytotoxicity (Figure S1) [21].

### 2.4. DNA Damage Detected by Comet Assays

Alkaline and neutral comet assays were performed after 24, 48, and 72 h, and the percentage of comet-formed cells reflected the level of DNA damage [22]. The alkaline assay detected both single- (SSB) and double-strand break (DSB), and the neutral assay detected DSB.

### 2.5. Determination of Cell Apoptosis

Cell apoptosis was analyzed after 72 h, using an annexin V assay (Nanjing KeyGen Biotech., Nanjing, China).

### 2.6. High Mobility Group Box 1 (HMGB1) in Culture Supernatants

Necrosis partly contributed to cell death in sonochemotherapy against cisplatin-resistant ovarian cancer cells [23]. Therefore, HMGB1 in the culture supernatants was quantified after 72 h to determine whether necrosis befell, using an enzyme-linked immunosorbent assay (Shino-Test, Kanagawa, Japan) [24].

### 2.7. Cell Death and Apoptosis after Using Inhibitors

Cell death and apoptosis occurred in only groups ZD2767P+CPG2 and ZD2767P+CPG2+US, and therefore, death modes were explored. The apoptosis inhibitor Z-VAD-fmk (Z-VAD; 10 *μ*M; Beyotime Biotechnol., Shanghai, China), ferroptosis inhibitor ferrostatin-1 (Fer; 2 *μ*M; MedChemExpress, Shanghai, China), or autophagy inhibitor 3-methyladenine (3MA; 1 mM; Selleck Chem., Houston, TX, USA) was added to medium after washing away ZD2767P and CPG2. Cell viability and apoptosis were determined after 72 h.

Intracellular reactive oxygen species (ROS) and iron were the pivotal molecules for ferroptosis [25, 26]. ROS was determined using the dichlorofluorescin diacetate assay (Beyotime). Fluorescent images were analyzed with the software ImageJ (NIH, Bethesda, MD, USA), and the mean density reflected the ROS level. Iron was determined with a colorimetric assay (Sigma-Aldrich, St. Louis, MO, USA).

### 2.8. Western Blot

Apoptosis proteins Bax, Bcl-2, and cleaved caspase 3 were determined after 72 h, using rabbit antibodies (Cell Signal. Technol., Danvers, MA, USA). The secondary antibody was a goat-anti-rabbit-IgG antibody (ZSGB-BIO, Beijing, China).

Ferroptosis modulins transferrin (TF), ferroportin (FPN), and glutathione peroxidase 4 (GPX4) and the autophagy protein microtubule-associated protein light chain 3 (LC3) were detected after 48 h. Antibodies against TF, FPN, and GPX4 were from Abcam (Cambridge, UK), and the anti-LC3 antibody was from Cell Signal. Technol. The secondary antibody was a goat-anti-rabbit-IgG antibody (Abcam).

*β*-Actin was the reference, using a rabbit antibody (Cell Signal. Technol.). Bands were assayed with the software Image Lab (Bio-Rad Lab., Hercules, CA, USA), and the level of a target protein was quantified with the density ratio.

### 2.9. Intracellular PK of ZD2767D

Intracellular PK of ZD2767D was explored. Considering the detection limit of the analytic method, 100, 200, or 400 *μ*M ZD2767P was adopted. Drugs were washed away 1 h after insonation, serum-free medium was added, and cells were maintained at 37°C till assays. The intracellular drug level was measured after 0 (immediately after insonation), 5, 15, 30, 60, and 90 min. Cells were homogenized to extract drugs. ZD2767D was determined using dual wavelength spectrophotometry, and the ZD2767D level was normalized by the protein concentration [13]. The ZD2767D level vs. time curve was analyzed to deduce the time to maximal level (T_max_), peak level (C_max_), area under the level vs. time curve from zero to last measurable time point (AUC_last_), and mean residence time from zero to last measurable level (MRT_last_), using the noncompartmental model [27]. Dose proportionality was evaluated by the quadratic regression model [28]. The percentage of cell uptake was calculated [13].

(1) $$\begin{array}{c} \text{cell uptake}=\frac{\text{maximum concentration}\times \text{homogenate volume}\times \text{dilution factor}}{\text{total amount of extracellular drug}}\times 100\% \end{array}$$

### 2.10. In Vivo Therapies

The animal trial was approved by the Institutional Review Board in compliance with the Guide for the Care and Use of Laboratory Animals. A549 or A549/DDP cells (1.0 × 10^7^) were subcutaneously injected in the scapular region of female BALB/c nude mice (aged 4–6 weeks; Ctr. Lab. Anim., Chongqing Med. Univ., Chongqing, China). When the tumor grew to 8 mm (about 2 weeks for A549 tumors and 3 weeks for A549/DDP tumors), mice were randomly divided into groups Ctrl, US, ZD2767P, ZD2767P+US, CPG2, CPG2+US, ZD2767P+CPG2, and ZD2767P+CPG2+US (5 mice per group). ZD2767P (10 mg/kg) was intravenously injected, CPG2 (0.12 U in 100 *μ*L) was intratumorally injected after 10 min, and then, the tumor was insonated (20 W/cm^2^ for 10 min) after 5 min [23]. Animals were euthanized after 14 days, and the volume and mass of a tumor were measured. Tumor tissues received pathological examinations, apoptosis was detected by an in situ end labeling assay (TUNEL; Servicebio Technol., Wuhan, China), and GPX4 was assayed by an immunohistochemical kit (ZSGB-BIO). Levels of apoptosis and GPX4 were quantified with the software ImageJ.

To determine whether CPG2 leaked into circulation, blood was sampled 30 min and 12 and 24 h after treatments. Serum CPG2 was determined using a kinetic assay [29]. Blood cell counts and biochemical analyses (alanine/aspartate aminotransferases, urea nitrogen, and creatinine) were performed at days 2 and 14, thereby assessing the safety.

### 2.11. Statistics

Data were processed with the software SAS (SAS Inst., Cary, NC, USA). Analysis of variance was used. The Student–Newman–Keuls test was used for multiple comparisons, and Dunnett's test was adopted when contrasting other groups with group Ctrl. The critical value was *p* < 0.05.

## 3. Results

### 3.1. ZD2767P+CPG2+US More Efficiently Deactivated Resistant NSCLC Cells

**Figure 1:**
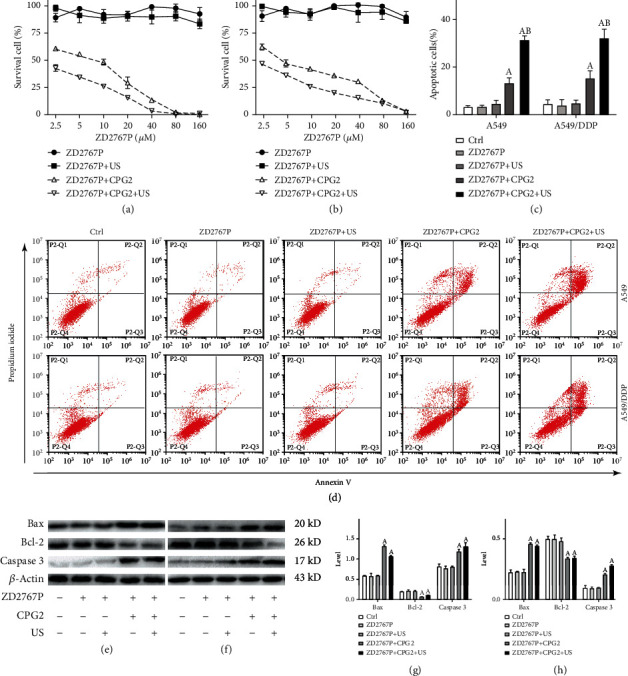
Cell death and apoptosis. Survival percentages of A549 (a) and A549/DDP (b) cells: ZD2767P+CPG2 and ZD2767P+CPG2+US decreased the survival percentage, with a lower value in the latter group. Apoptosis (c, d): ZD2767P+CPG2 and ZD2767P+CPG2+US caused apoptosis, and more apoptotic cells were detected in the latter group. Apoptosis proteins in A549 (e, g) and A549/DDP (f, h) cells: levels of Bax and cleaved caspase 3 were increased in groups ZD2767P+CPG2 and ZD2767P+CPG2+US, but that of Bcl-2 was decreased. Data were mean ± standard deviation for 3 independent experiments. A: vs. Ctrl, *p* < 0.05; B: vs. ZD2767P+CPG2, *p* < 0.05.

No cell death occurred in groups ZD2767P and ZD2767P+US, indicating that ZD2767P was noncytotoxic and that ultrasound did not activate ZD2767P; the cell survival percentage was decreased in groups ZD2767P+CPG2 and ZD2767P+CPG2+US, with a lower value in the latter group (A549: *p* < 0.0001; A549/DDP: *p* < 0.0001) (Figures 1(a) and 1(b)). The cell survival percentage in group ZD2767P+CPG2 or ZD2767P+CPG2+US was ≥25% at 10 *μ*M ZD2767P, and this level was adopted in following trials. ZD2767P+CPG2+US led to a lower IC_50_ in comparison with ZD2767P+CPG2, with values of 3.0 vs. 6.4 *μ*M in A549 cells and 2.9 vs. 6.1 *μ*M in A549/DDP cells, respectively. CI was 1.20 (0.99–1.40) in A549 cells and 1.16 (1.01–1.29) in A549/DDP cells, i.e., insonation synergized ZD2767P+CPG2. These data demonstrated that ZD2767P+CPG2+US was more effective to deactivate cisplatin-resistant NSCLC cells.

### 3.2. ZD2767P+CPG2+US Directly Caused DSB

**Figure 2:**
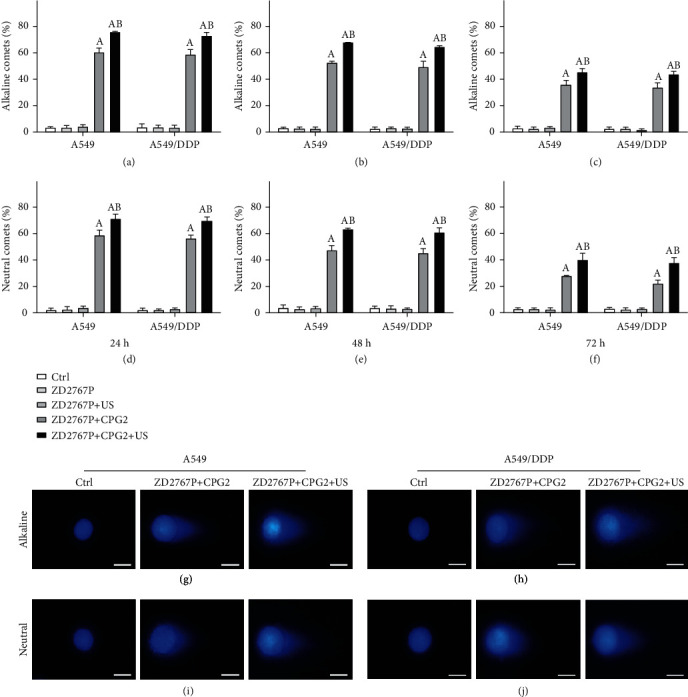
DNA damage detected by the comet assays. Alkaline (a–c, g, h) and neutral (d–f, i, j) assays at 24, 48, and 72 h: ZD2767P+CPG2 and ZD2767P+CPG2+US led to comet formation, with a higher comet percentage in the latter group; the comet percentage gradually decreased after 24 h, indicating DNA repair; the neutral-comet percentage approached to the alkaline-comet one, demonstrating that DSB was the major mode of DNA damage. The scale bar was 50 *μ*m. Data were mean ± standard deviation for 3 independent experiments. A: vs. Ctrl, *p* < 0.05; B: vs. ZD2767P+CPG2, *p* < 0.05.

The percentage of comet-formed cells was increased in only group ZD2767P+CPG2 or ZD2767P+CPG2+US at 24–72 h, with a higher value in the latter group, in either alkaline or neutral assay (A549: *p* < 0.0001 for each; A549/DDP: *p* < 0.0001 for each). A downtrend was noted after 24 h, confirming DNA repair occurred. Alkaline-comet percentages in A549 and A549/DDP cells were nearly equal in group ZD2767P+CPG2 or ZD2767P+CPG2+US (*p* = 0.0543–0.6244, *p* = 0.0563–0.2185). This finding was also verified in the neutral assay (*p* = 0.0764–0.0904, *p* = 0.0620–0.2683), with an exception of group ZD2767P+CPG2 at 48 h (47.5 ± 3.3% vs. 45.4 ± 3.1%, *p* = 0.0378). The neutral-comet percentage approached to the alkaline-comet one, demonstrating that DSB was the major mode of DNA damage (Figure 2). These data indicated that ZD2767P+CPG2+US directly induced DSB.

### 3.3. ZD2767P+CPG2+US Induced More Cells to Undergo Apoptosis

Neither ZD2767P nor ZD2767P+US caused apoptosis, according to data on cell death and DNA damage. The apoptosis percentage was increased in groups ZD2767P+CPG2 and ZD2767P+CPG2+US, with a higher level in the latter group (A549: *p* < 0.0001; A549/DDP: *p* < 0.0001) (Figures 1(c) and 1(d)). ZD2767P+CPG2 led to similar apoptosis percentages in A549 and A549/DDP cells (*p* = 0.1818), so did ZD2767P+CPG2+US (*p* = 0.6797).

Western blot indicated upregulation of Bax and caspase 3 in groups ZD2767P+CPG2 and ZD2767P+CPG2+US, whereas Bcl-2 was downregulated (A549: *p* < 0.0001 for each; A549/DDP: *p* < 0.0001 for each) (Figures 1(e)–1(h)). The HMGB1 level was not altered (A549: *p* = 0.5187; A549/DDP: *p* = 0.1075), confirming no cell necrosis occurred (Figure S2). These data manifested that ZD2767P+CPG2+US resulted in more apoptotic cells.

### 3.4. Ferroptosis Played a Part in Cell Death due to ZD2767P+CPG2+US

**Figure 3:**
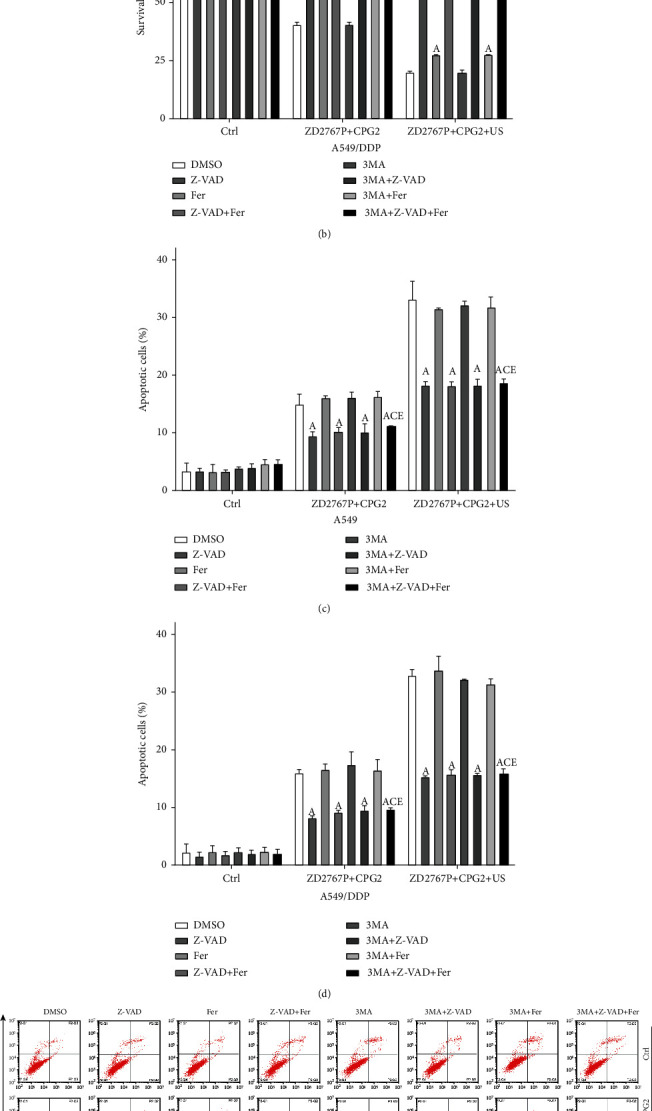
Cell survival and apoptosis in groups ZD2767P+CPG2 and ZD2767P+CPG2+US after using inhibitors. Survival percentages of A549 (a) and A549/DDP (b) cells: Z-VAD or Fer increased the survival percentage, and the highest percentage was noted when using both agents; 3MA did not affect cell death. Apoptosis (c–e): Z-VAD decreased the apoptosis percentage, but Fer/3MA did not affect apoptosis. Data were mean ± standard deviation for 3 independent experiments. A: vs. Ctrl, *p* < 0.05; B: vs. Z-VAD, *p* < 0.05; C: vs. Fer, *p* < 0.05; D: vs. 3MA+Z-VAD, *p* < 0.05; E: vs. 3MA+Fer, *p* < 0.05.

Cell death modes in groups ZD2767P+CPG2 and ZD2767P+CPG2+US were explored. The apoptosis percentage was far less than the cell death one, suggesting that there be other cell death modes. Z-VAD or Fer increased the cell survival percentage, and the highest value occurred when using both inhibitors; 3MA did not affect the survival percentage (A549: *p* < 0.0001 for each; A549/DDP: *p* < 0.0001 for each) (Figures 3(a) and 3(b)). Z-VAD alone or combined with Fer/3MA decreased the apoptosis percentage, but Fer or 3MA alone did not impact on apoptosis (A549: *p* < 0.0001 for each; A549/DDP: *p* < 0.0001 for each) (Figures 3(c)–3(e)).

**Figure 4:**
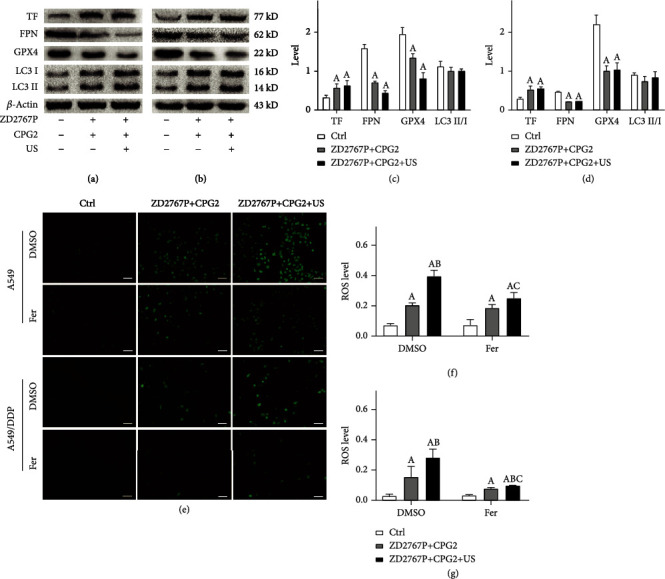
Ferroptosis/autophagy proteins and intracellular ROS in groups ZD2767P+CPG2 and ZD2767P+CPG2+US. Proteins in A549 (a, c) and A549/DDP (b, d) cells: the TF level was increased, and levels of FPN and GPX4 were decreased; the LC3 II/I level was unaltered. ROS fluorescent images (e). ROS levels in A549 (f) and A549/DDP (g) cells: the level was increased after treatments, with a higher level in the latter group; Fer reduced the ROS level in group ZD2767P+CPG2+US. The scale bar was 50 *μ*m. Data were mean ± standard deviation for 3 independent experiments. A: vs. Ctrl, *p* < 0.05; B: vs. ZD2767P+CPG2, *p* < 0.05; C: vs. the same group under DMSO, *p* < 0.05.

Ferroptosis proteins were assayed. The level of TF was increased (A549: *p* = 0.0132; A549/DDP: *p* = 0.0042), and those of FPN and GPX4 were decreased (A549: *p* < 0.0001 for each; A549/DDP: *p* < 0.0001 for each). The LC3 II/I level was not altered; i.e., no autophagy occurred (A549: *p* = 0.2607; A549/DDP: *p* = 0.0640) (Figures 4(a)–4(d)).

The level of intracellular ROS in group ZD2767P+CPG2 or ZD2767P+CPG2+US was increased (A549: *p* = 0.0003; A549/DDP: *p* = 0.0075). Fer reduced the ROS level in group ZD2767P+CPG2+US (A549: *p* = 0.0379; A549/DDP: *p* = 0.0315) (Figures 4(e)–4(g)). A higher iron level was noted (A549: *p* = 0.0007; A549/DDP: *p* = 0.0191) (Figure S3). These data demonstrated that ZD2767P+CPG2+US deactivated cells via apoptosis and ferroptosis pathways.

### 3.5. ZD2767P+CPG2+US Led to Higher C_max_, AUC_last_, and MRT_last_ of ZD2767D

ZD2767D was detected in only groups ZD2767P+CPG2 and ZD2767P+CPG2+US. Higher C_max_, AUC_last_, and MRT_last_ were noted in group ZD2767P+CPG2+US (C_max_: 1.6–2.0 fold, *p* = 0.0006–0.0382; AUC_last_: 1.8–3.5 fold, *p* < 0.0001 to 0.0066; MRT_last_: 1.1–1.9 fold, *p* < 0.0001 to 0.0274); the cell uptake percentage was 7.4 ± 0.9% vs. 4.1 ± 0.3% (*p* < 0.0001) (Figure S4 and Table 1). Neither C_max_ nor AUC_last_ ratio accorded with the dose ratio (1 : 2 : 4); the regression model demonstrated PK proportionality in group ZD2767P+CPG2, but nonproportionality in group ZD2767P+CPG2+US (Table S1). These data indicated that ZD2767P+CPG2+US led to higher C_max_, AUC_last_, and MRT_last_ of ZD2767D compared with ZD2767P+CPG2.

### 3.6. ZD2767P+CPG2+US Produced Stronger Antitumor Action and Induced Ferroptosis In Vivo

**Figure 5:**
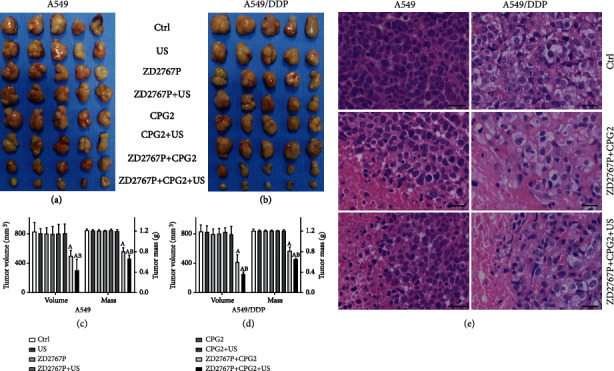
Treatments of xenograft tumors. Tumor volume and mass (a–d): ZD2767P+CPG2 and ZD2767P+CPG2+US inhibited tumors. Pathological images (e): tumor necrosis was noted after treatments. The scale bar was 50 *μ*m. Data were mean ± standard deviation for 5 mice. A: vs. Ctrl, *p* < 0.05; B: vs. ZD2767P+CPG2, *p* < 0.05.

The tumor volume in group US, ZD2767P, ZD2767P+US, CPG2, or CPG2+US did not differ from that in group Ctrl, i.e., no antitumor effect; the volume was shrunk in groups ZD2767P+CPG2 and ZD2767P+CPG2+US, with a smaller value in the latter group (A549: *p* < 0.0001, A549/DDP: *p* < 0.0001); the inhibitory rates in groups ZD2767P+CPG2 and ZD2767P+CPG2+US were 39.7% and 63.5% in A549 tumors and 50.0% and 70.1% in A549/DDP tumors, respectively. The mass inhibitory rates were 33.9% and 45.5% in A549 tumors and 34.3% and 46.6% in A549/DDP tumors, respectively (Figure 5).

**Figure 6:**
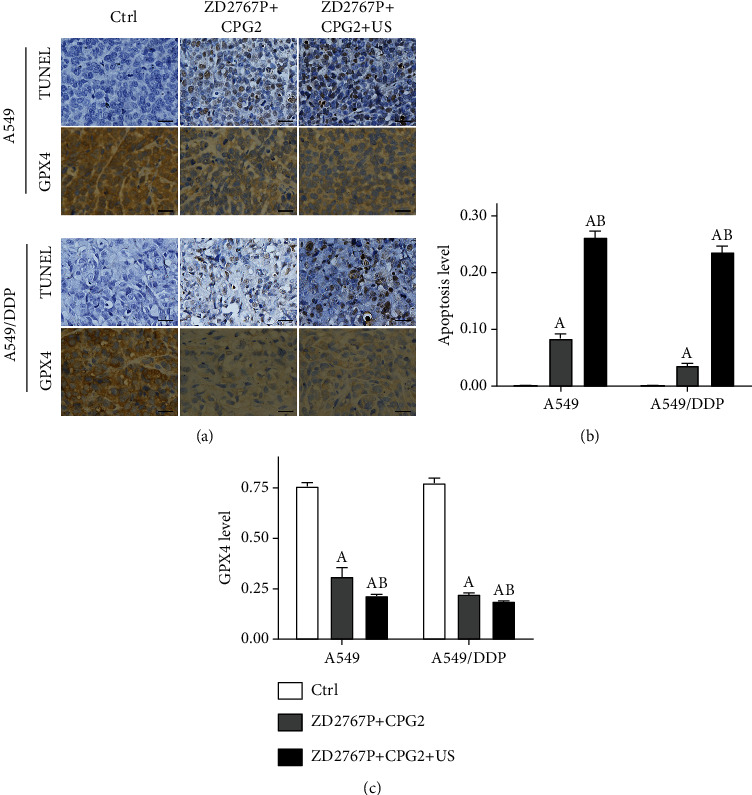
Apoptosis (TUNEL) and GPX4 in tumor tissues. Representative images (a). Expression levels (b, c): the apoptosis level was increased in groups ZD2767P+CPG2 and ZD2767P+CPG2+US, whereas the GPX4 level was reduced. The scale bar was 50 *μ*m. Data were mean ± standard deviation for 5 mice. A: vs. Ctrl, *p* < 0.05; B: vs. ZD2767P+CPG2, *p* < 0.05.

In groups ZD2767P+CPG2 and ZD2767P+CPG2+US, the apoptosis level (TUNEL) in tumor tissues was increased, but the GPX4 level was decreased (A549: *p* < 0.0001 for each; A549/DDP: *p* < 0.0001 for each) (Figure 6 and S5).

No serum CPG2 was detected. No severe safety events occurred. An increase in serum alanine aminotransferase in group ZD2767P+CPG2+US, and a slight decrease in erythrocyte count (within the normal range) in groups ZD2767P+CPG2 and ZD2767P+CPG2+US were noted at day 14 in mice bearing A549 tumors (Tables S2 and S3). These data indicated that ZD2767P+CPG2+US produced stronger in vivo anticancer action with good tolerability and that ZD2767P+CPG2+US induced apoptosis and ferroptosis in tumors.

## 4. Discussion

Apoptosis insufficiency resulted in chemoresistance in A549/DDP cells [17]. Here, the apoptosis percentage in A549/DDP cells was almost equal to that in A549 cells, indicating that ZD2767D bypassed the cisplatin-apoptosis pathway to cause apoptosis in resistant cells. In groups ZD2767P+CPG2 and ZD2767P+CPG2+US, the apoptosis percentage was far less than the cell death one, suggesting that there be other cell death modes. HMGB1 was assayed since necrosis played a part in ultrasonic chemosensitization on resistant ovarian cancer cells COC1/DDP [23]. HMGB1 was unaffected in this study. Therefore, nonapoptotic death due to ZD2767P+CPG2+US was not necrosis and should be elucidated.

ZD2767D insulted DNA to cause DNA break [3–5]. Comet percentages in A549 and A549/DDP cells were close, being consistent with the apoptosis data. The comet percentage in the alkaline assay approached to that in the neutral assay, indicating that DSB was the major mode of DNA damage (i.e., ZD2767D directly induced DSB). ZD2767D caused interstrand crosslinks [3]. Cisplatin frequently created intrastrand crosslinks to cause SSB, and a part of SSB would evolve into DSB; cisplatin can induce interstrand crosslinks to cause DSB; unrepairable DSB led to cell death via apoptosis or necrosis [30–32]. SSB was repaired via NER, DSB was repaired via HR, and resistant cells had an improved capacity of DNA repair [14, 15, 32]. Directly inducing DSB implied that a higher NER capacity cannot protect cells when exposed to ZD2767P+CPG2 or ZD2767P+CPG2+US. NER played an important part in resistance of NSCLC, and therefore, the response of A549/DDP cells was similar to that of A549 cells [2, 16]. The DSB repair should be elucidated to thoroughly understand action of the ZD2767P+CPG2 or ZD2767P+CPG2+US modality.

Cell death and apoptosis in the presence of inhibitors were explored to elucidate the death modes in ZD2767P+CPG2 and ZD2767P+CPG2+US treatments. The cell survival percentage was increased when using Z-VAD or Fer, and the highest value was noted when combining these two inhibitors. The survival percentage in the presence of Z-VAD was higher than that in the presence of Fer. 3MA did not affect the survival percentage and not modulate cell protection due to Z-VAD or Fer. These data indicated that ZD2767P+CPG2 or ZD2767P+CPG2+US deactivated cells via apoptosis and ferroptosis pathways and that apoptosis had a higher weight. Z-VAD decreased the apoptosis percentage, and Fer or 3MA did not affect apoptosis protection due to Z-VAD. Thus, both apoptosis and ferroptosis were triggered in ZD2767P+CPG2 or ZD2767P+CPG2+US treatments. Western blot demonstrated upregulation of TF and downregulation of FPN and GPX4. This led to iron overload in cells, since TF mediated the influx of iron and FPN mediated the efflux [33]. GPX4 reduced iron-dependent hydroperoxides being the key molecule of ferroptosis, and glutathione was a cofactor of GPX4 [34, 35]. Insonation decreased the level of glutathione in resistant cells COC1/DDP, which can reduce the activity of GPX4 [23, 35]. The iron accumulation, GPX4 decrease, and ROS caused lipid peroxidation, leading to ferroptosis [25, 36]. Fer prevented ferroptosis via inhibiting lipid peroxidation [25]. These were consistent with the present data that a higher ROS level was detected after treatments and that Fer reduced the ROS level. Signals of DNA damage were transmitted to mitochondria, regulating Bcl-2/Bax to initiate apoptosis [30, 31]. Mitochondria generated ROS that participated in apoptosis and ferroptosis [25, 26]. DNA damage responses that involved in DNA repair and apoptosis can induce ferroptosis: ATM (ataxia-telangiectasia mutated) regulated ferroptosis via MTF1 (metal regulatory transcription factor 1) and iron metabolisms; p53 affected ferroptosis via SLC7A11 (glutamate/cystine antiporter solute carrier family 7 member 11), ACSL4 (long chain acyl-coenzyme A synthetase 4), ALOX12 (arachidonate 12-lipoxygenase), and DPP4 (dipeptidyl peptidase 4); MDM2/MDMX (mouse double minute) modulated ferroptosis via FSP1 (ferroptosis suppressor protein 1) and coenzyme Q10 [37]. Therefore, severe DSB simultaneously triggered apoptosis and ferroptosis. Ferroptosis offset the apoptosis deficiency, thereby deactivating plenty of resistant cells.

No ZD2767D in cells in groups ZD2767P and ZD2767P+US manifested that ultrasound alone cannot activate ZD2767P, which was consistent with data on ovarian cancer cells [13]. A higher C_max_ and a longer MRT were noted in group ZD2767P+CPG2+US compared with group ZD2767P+CPG2, which increased AUC. C_max_ and AUC determined the efficacy of a drug, so ZD2767P+CPG2+US produced stronger antitumor actions [38, 39]. Active CPG2 cannot enter cells even under insonation because of a molecular mass of 83 kD [12, 13]. Hence, intracellular ZD2767D was only from the extracellular medium. A low cell uptake percentage indicated that ZD2767D crossed cell membranes via passive diffusion: the influx rate was reliant on the concentration gradient between extracellular and intracellular media. Insonation boosted the specific activity of CPG2, thereby improving the yield rate of ZD2767D to increase the extracellular concentration during an early period (i.e., a higher gradient) [13]. Insonation enhanced passive diffusion and permeabilized cell membranes to prolong the duration time of drug influx [10]. These effects increased intracellular amount of ZD2767D, thereby elevating C_max_ and AUC. MRT of ZD2767D in cells (23 min in group ZD2767P+CPG2 and 30 min in group ZD2767P+CPG2+US) was far longer than its half-life in plasma (≈2 min) that played a part in low antitumor efficacy, suggesting that transferring ZD2767D into cells can prolong the in vivo retention time [3, 5, 19].

PK of ZD2767D was proportional in group ZD2767P+CPG2, but was nonproportional in group ZD2767P+CPG2+US. These findings were inconsistent with data on ovarian cancer cells SKOV3 and SKOV3/DDP, where nonproportionality was verified [13]. These results indicated that dose proportionality depended on the drug and cell type. Ultrasonic therapy operated in the range of nonlinear acoustics, i.e., membrane permeabilization did not increase linearly. Thus, the drug influx was not increased proportionally. Nonproportional PK should be considered when planning a therapy.

In vivo data demonstrated the efficacy of ZD2767P+CPG2 or ZD2767P+CPG2+US, and ZD2767P+CPG2+US produced a better therapeutic outcome. Enough amount of ZD2767D was generated within the tumor before insonation, since there was an interval between drug injection and insonation. Insonation impelled more ZD2767D molecules to flow into cells, thereby increasing the intracellular amount and the in vivo retention time. Intratumoral injection of CPG2 to a certain extent confined the ZD2767P activation to the tumor, i.e., realizing a targeted treatment. Limited by a short half-life of ZD2767D and the sensitivity of spectrophotometry, the serum ZD2767D level was not quantified in this study. The leakage of ZD2767D (including that due to extratumroal activation of ZD2767P) can be assessed with toxicities to normal tissues [19]. Here, no safety issues manifested a few leakages. A short half-life commonly implied less toxicity. Severe toxicities reported in previous clinical trials may be due to the persistent release of ZD2767D, because CPG2 was intravenously injected leading to diffusive distribution [8, 19]. Insonation extended the intratumoral distribution of CPG2 to enlarge the production field of ZD2767D, i.e., more segments were treated [13]. These effects improved the antitumor efficacy. The pattern of TUNEL and GPX4 indicated that ZD2767P+CPG2 deactivated cells via inducing apoptosis and ferroptosis [40]. Both were increased in group ZD2767P+CPG2+US, thereby leading to a better therapeutic outcome. A small injection volume reduced the leakage of CPG2 from tumors avoiding off-target activation of ZD2767P, according to the safety data—no serum CPG2 was detected and no severe systemic toxicities were observed.

Limited by the sensitivity of the analytic method, the ZD2767P dose in PK trials was higher than that in therapies, and the ZD2767D level in vivo was not calibrated. PK data should be considered a reference to understand the PK property of ZD2767P+CPG2+US. Ultrasound had good tissue penetrate ability and can be focused on the tumor under image guidance without harming tissues lying in the traveling path of ultrasound [10, 41]. These can further improve the therapeutic precision because ZD2767P+CPG2 was a targeted therapy. Therefore, ZD2767P+CPG2+US can realize precise treatments, where the mode of CPG2 administration should be optimized to match insonation. Here demonstrated ferroptosis in vitro and in vivo. ZD2767P+CPG2+US should be thoroughly elucidated since ferroptosis was a promising therapeutic target for lung cancer [42]. So far data revealed that sensitive and resistant lung/ovary cancer cells responded to ZD2767P+CPG2+US similarly. Resistance mechanisms varied between cancer types; i.e., ZD2767P+CPG2+US should be tested on more cancer types to determine types and subtypes that can benefit from this means. Whether the present results can be extended to antibody-directed enzyme prodrug therapy should be addressed, particularly considering that intravenously infusing antibody-linked CPG2 had low targeting efficacy (the level ratio of tumor to serum was 0.4) [19].

## 5. Conclusion

Either ZD2767P+CPG2 or ZD2767P+CPG2+US was effective against cisplatin-resistant NSCLC cells, and ZD2767P+CPG2+US produced a better therapeutic outcome in vitro and in vivo. ZD2767P+CPG2+US led to higher C_max_, AUC, and MRT of ZD2767D. Apoptosis and ferroptosis were the cell death pathways. Therefore, ZD2767P+CPG2+US was a candidate therapy for resistant NSCLC.

## Figures and Tables

**Table 1:** Intracellular PK parameters of ZD2767D.

	A549		A549/DDP	
	ZD2767P+CPG2	ZD2767P+CPG2+US	ZD2767P+CPG2	ZD2767P+CPG2+US
*100 μM*				
T_max_ (min)^a^	5	5	5	5
C_max_ (nmol/mg)	3.89 ± 0.13	7.42 ± 0.93^c^	3.96 ± 0.23	7.83 ± 1.12^c^
AUC_last_ (nmol∙min/mg)	101.91 ± 4.78	343.62 ± 29.46^c^	104.12 ± 5.27	369.00 ± 33.49^c^
MRT_last_ (min)	15.01 ± 0.27	28.67 ± 0.29^c^	14.91 ± 0.32	28.86 ± 0.42^c^
Uptake (%)	4.25 ± 0.33	7.95 ± 0.77^c^	4.47 ± 0.43	8.70 ± 0.89^c^
*200 μM*				
T_max_ (min)^a^	5	5	5	5
C_max_ (nmol/mg)	6.16 ± 0.89	10.82 ± 0.66^c^	6.29 ± 0.56	11.23 ± 0.87^c^
AUC_last_ (nmol∙min/mg)	268.16 ± 33.63	484.94 ± 20.56^c^	277.48 ± 39.43	489.73 ± 41.86^c^
MRT_last_ (min)	25.40 ± 0.30	29.04 ± 0.36^c^	25.25 ± 1.16	28.97 ± 0.10^c^
Uptake (%)	4.20 ± 0.62	7.58 ± 0.35^c^	4.23 ± 0.41	7.64 ± 0.70^c^
*400 μM*				
T_max_ (min)^a^	15	15	15	15
C_max_ (nmol/mg)	13.34 ± 0.22	20.88 ± 0.55^c^	12.47 ± 0.70	20.72 ± 0.35^c^
AUC_last_ (nmol∙min/mg)	521.49 ± 17.90	1084.90 ± 5.80^c^	487.76 ± 25.19	1067.85 ± 16.86^c^
MRT_last_ (min)	27.25 ± 0.51	32.50 ± 0.07^c^	27.27 ± 0.16	32.43 ± 0.10^c^
Uptake (%)	3.96 ± 0.06	6.38 ± 0.01^c^	3.71 ± 0.13	6.41 ± 0.20^c^
*Ratio* ^b^				
C_max_	1.0 : 1.6 : 3.4	1.0 : 1.5 : 2.8	1.0 : 1.6 : 3.2	1.0 : 1.4 : 2.6
AUC	1.0 : 2.6 : 5.1	1.0 : 1.4 : 3.2	1.0 : 2.7 : 4.7	1.0 : 1.3 : 2.9

^a^: statistic comparison was not applicable; ^b^: value at 100 *μ*M was the reference when calculating the ratio; ^c^: vs. without US at the same dose, *p* < 0.05.

## Data Availability

The data used to support the findings of this study are included within the article.

## Supplementary Materials

Supplementary Materials Figure S1: cell death and apoptosis after exposure to US and/or CPG2. Figure S2: HMGB1 level in culture supernatants. Figure S3: level of intracellular iron in groups ZD2767P+CPG2 and ZD2767P+CPG2+US. Figure S4: level vs. time curve of ZD2767D in groups ZD2767P+CPG2 and ZD2767P+CPG2+US. Figure S5: TUNEL and GPX4 expressions in tumor tissues. Table S1: assessment of dose proportionality using the quadratic regression model. Table S2: body mass and hematological/biochemical parameters in mice bearing A549 tumors. Table S3: body mass and hematological/biochemical parameters in mice bearing A549/DDP tumors.
