# Supplementary material for: Ultrasonically Enhanced ZD2767P–Carboxypeptidase G2 Deactivates Cisplatin-Resistant Human Lung Cancer Cells
Source: Oxid Med Cell Longev. 2022 Nov 4;2022:9191233. doi: 10.1155/2022/9191233 (PMC9652066; doi:10.1155/2022/9191233)
Supplement: Supplementary Materials — Figure S1: cell death and apoptosis after exposure to US and/or CPG2. Figure S2: HMGB1 level in culture supernatants. Figure S3: level of intracellular iron in groups ZD2767P+CPG2 and ZD2767P+CPG2+US. Figure S4: level vs. time curve of ZD2767D in groups ZD2767P+CPG2 and ZD2767P+CPG2+US. Figure S5: TUNEL and GPX4 expressions in tumor tissues. Table S1: assessment of dose proportionality using the quadratic regression model. Table S2: body mass and hematological/biochemical parameters in mice bearing A549 tumors. Table S3: body mass and hematological/biochemical parameters in mice bearing A549/DDP tumors. [file 9191233.f1.docx]

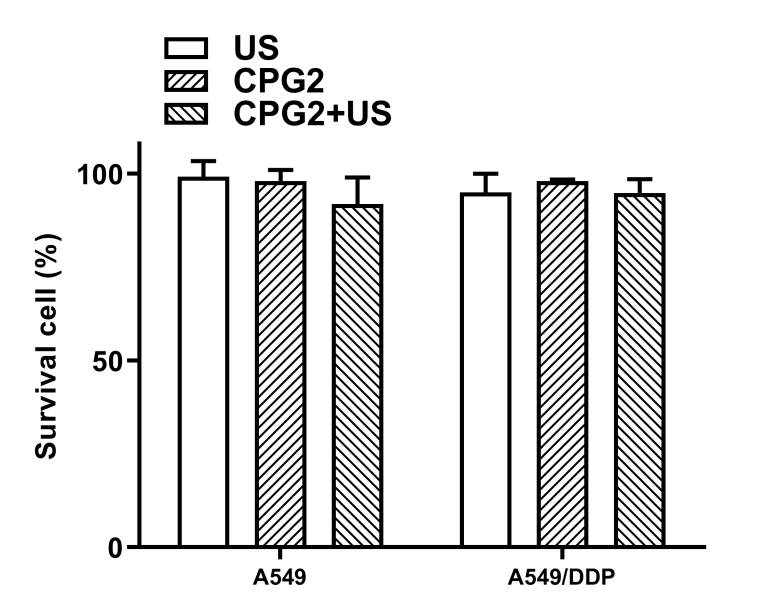


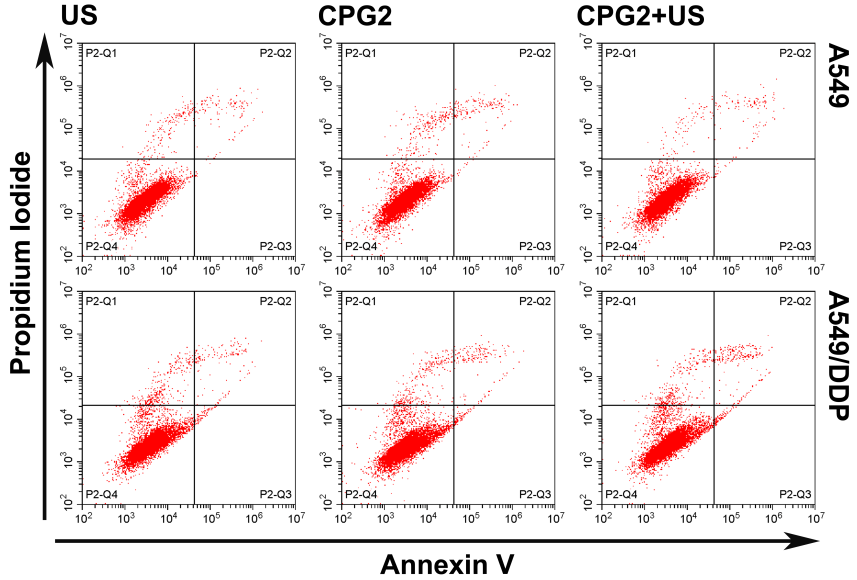


**Figure S1.** Cell death (*upper*) and apoptosis (*down*) after exposure to US (10 W/cm^2^, 20 s) and/or CPG2 (1.2 U/ml) (n = 3). No cytotoxicity was detected.


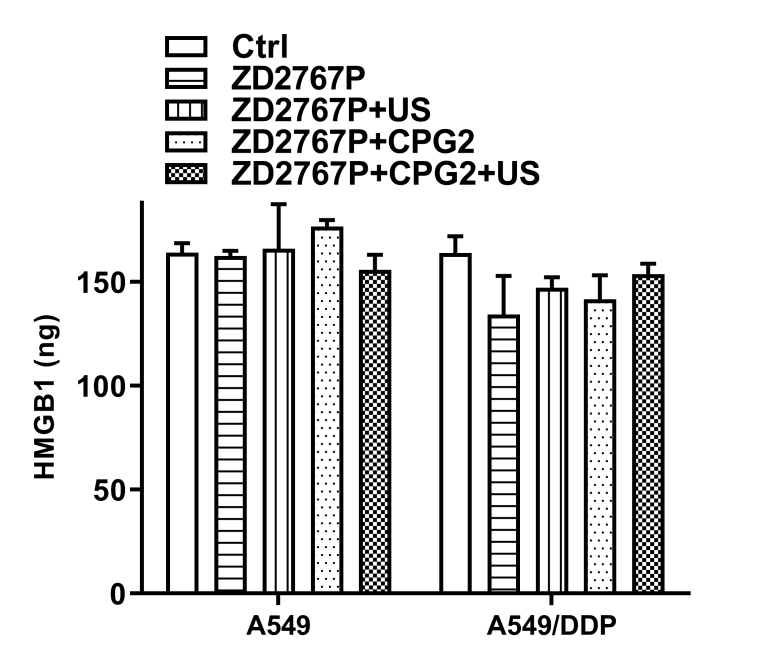


**Figure S2.** HMGB1 level in culture supernatants (n = 3). No increase demonstrated no cell necrosis occurred.

**
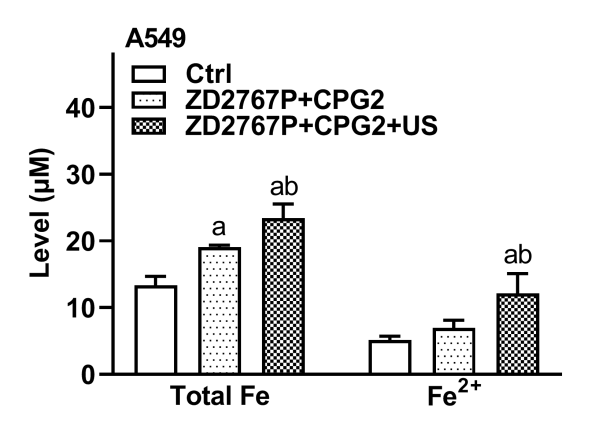

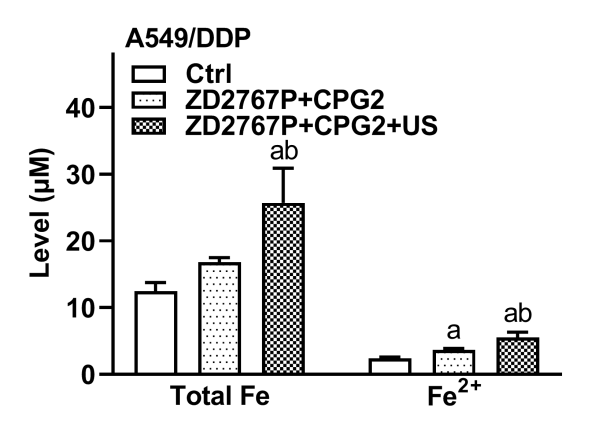
**

**Figure S3.** Level of intracellular iron in groups ZD2767P+CPG2 and ZD2767P+CPG2+US (n = 3). a: vs. Ctrl, p < 0.05; b: vs. ZD2767P+CPG2, p < 0.05.

**
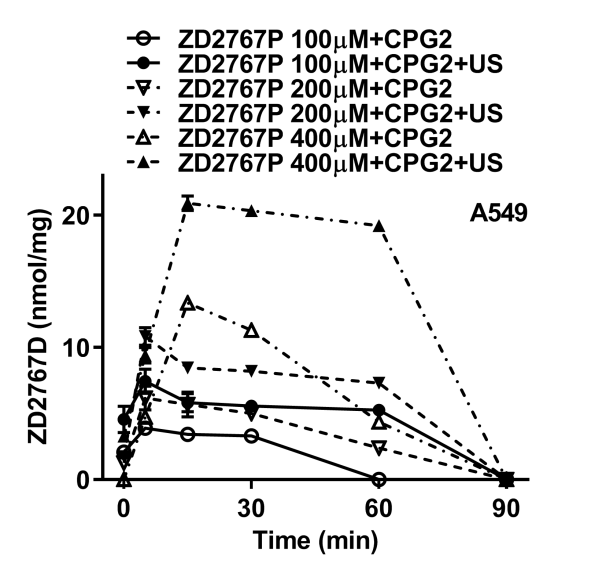

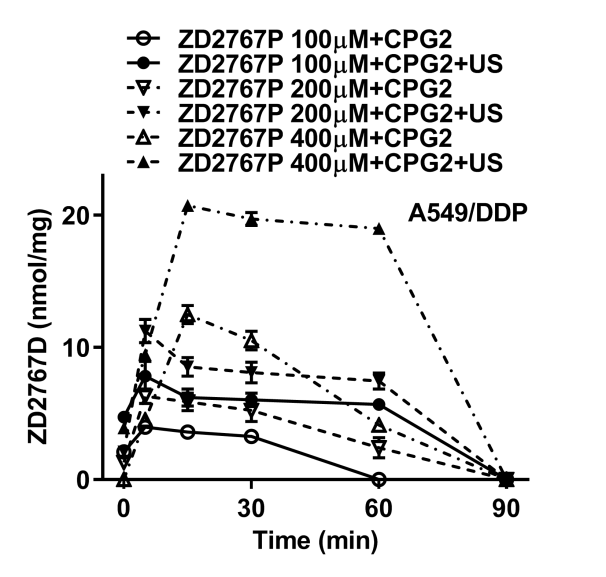
**

**Figure S4.** Level vs. time curves of ZD2767D in groups ZD2767P+CPG2 and ZD2767P+CPG2+US (n = 3).

**Table S1.** Assessment of dose proportionality using the quadratic regression model

|  | ZD2767P+CPG2 | | ZD2767P+CPG2+US | |
| --- | --- | --- | --- | --- |
|  | C_max_ | AUC_last_ | C_max_ | AUC_last_ |
| A549 | + | + | ‒ | ‒ |
| A549/DDP | + | + | ‒ | ‒ |

+: proportional; ‒: nonproportional.


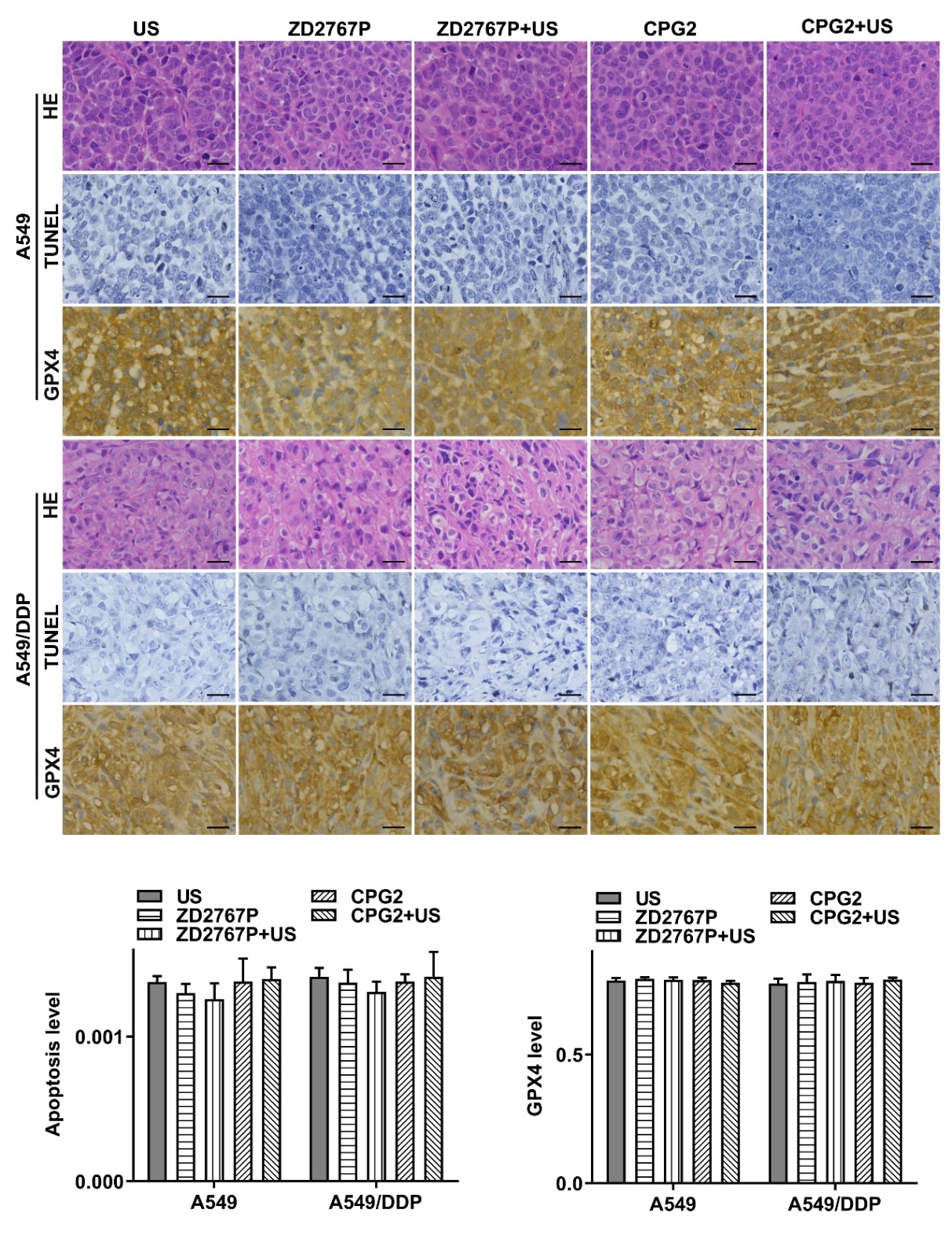


**Figure S5.** TUNEL and GPX4 expression in tumor tissues (n = 5). Apoptosis (TUNEL) and GPX4 levels were not altered in group US, ZD2767P, ZD2767P+US, CPG2 or CPG2+US. The scale bar was 50 μm.

**Table S2.** Body mass and hematological/biochemical parameters in mice bearing A549 tumors (n = 3−5)

|  | Day 2 | | | | | | | Day 14 | | | | | | | |
| --- | --- | --- | --- | --- | --- | --- | --- | --- | --- | --- | --- | --- | --- | --- | --- |
|  | RBC  (10^12^/L) | WBC  (10^9^/L) | Pt  (10^9^/L) | ALT  (U/L) | AST  (U/L) | Cr  (μM) | BUN  (mM) | RBC  (10^12^/L) | WBC  (10^9^/L) | Pt  (10^9^/L) | ALT  (U/L) | AST  (U/L) | Cr  (μM) | BUN  (mM) | BM  (g) |
| Ctrl | 9.76±  0.29 | 7.06±  1.06 | 758±  40 | 57±  3 | 129±  4 | 14.4±  2.8 | 7.6±  0.2 | 10.84±  0.71 | 6.40±  2.11 | 679±  162 | 39±  3 | 139±  25 | 12.7±  1.2 | 10.8±  0.8 | 20.5±  0.6 |
| US | 10.16±  0.23 | 5.76±  0.71 | 772±  20 | 61±  2 | 173±  23 | 13.7±  0.9 | 8.7±  0.9 | 9.38±  0.16*^a^* | 4.04±  1.18 | 646±  108 | 37±  6 | 107±  28 | 12.6±  0.4 | 9.0±  3.2 | 19.5±  1.1 |
| ZD2767P | 10.03±  0.16 | 5.11±  0.46 | 733±  53 | 58±  11 | 141±  25 | 12.6±  1.3 | 8.2±  2.1 | 9.96±  0.41 | 4.30±  2.89 | 612±  203 | 39±  6 | 125±  30 | 13.7±  2.0 | 7.8±  1.4 | 19.7±  1.0 |
| ZD2767P+US | 9.92±  0.06 | 4.55±  0.22 | 730±  62 | 50±  14 | 133±  38 | 14.4±  2.5 | 9.3±  2.7 | 9.67±  0.23*^a^* | 7.94±  3.41 | 909±  177 | 40±  8 | 95±  13 | 18.7±  0.5*^a^* | 7.4±  1.1 | 19.2±  1.1 |
| CPG2 | 10.44±  0.43 | 4.72±  1.08 | 680±  75 | 46±  9 | 120±  32 | 11.4±  2.4 | 8.7±  1.1 | 10.25±  0.25 | 6.32±  1.19 | 724±  149 | 50±  6 | 138±  19 | 13.5±  1.7 | 7.1±  0.5 | 19.9±  0.6 |
| CPG2+US | 10.13±  0.57 | 6.70±  3.62 | 864±  154 | 49±  17 | 158±  42 | 13.4±  1.9 | 7.4±  1.9 | 10.30±  0.49 | 5.48±  2.49 | 731±  36 | 54±  2 | 173±  4 | 12.0±  2.5 | 7.1±  1.0 | 20.4±  0.5 |
| ZD2767P+CPG2 | 10.64±  0.52*^a^* | 8.42±  1.06 | 924±  23 | 46±  6 | 121±  12 | 12.1±  2.1 | 10.7±  4.2 | 9.57±  0.47*^a^* | 5.17±  1.08 | 617±  95 | 54±  11 | 166±  21 | 15.3±  3.0 | 6.0±  2.1*^a^* | 18.5±  0.4*^a^* |
| ZD2767P+CPG2+US | 10.31±  0.35 | 5.65±  1.86 | 736±  102 | 59±  5 | 133±  6 | 13.8±  2.0 | 10.5±  1.6 | 9.55±  0.48*^a^* | 6.90±  1.97 | 636±  96 | 85±  16*^a^* | 179±  9 | 14.9±  3.9 | 6.7±  0.8*^a^* | 18.2±  0.5*^a^* |

RBC: red blood cell; WBC: white blood cell; Pt: platelet; Cr: creatinine; BUN: urea nitrogen; ALT: alanine aminotransferase; AST: aspartate aminotransferase; BM: body mass; *^a^*: vs. Ctrl: p < 0.05.

**Table S3.** Body mass and hematological/biochemical parameters in mice bearing A549/DDP tumors (n = 3−5)

|  | Day 2 | | | | | | | Day 14 | | | | | | | |
| --- | --- | --- | --- | --- | --- | --- | --- | --- | --- | --- | --- | --- | --- | --- | --- |
|  | RBC  (10^12^/L) | WBC  (10^9^/L) | Pt  (10^9^/L) | ALT  (U/L) | AST  (U/L) | Cr  (μM) | BUN  (mM) | RBC  (10^12^/L) | WBC  (10^9^/L) | Pt  (10^9^/L) | ALT  (U/L) | AST  (U/L) | Cr  (μM) | BUN  (mM) | BM  (g) |
| Ctrl | 11.14±  0.42 | 6.69±  3.51 | 840±  135 | 54±  3 | 149±  9 | 12.6±  2.4 | 12.5±  0.5 | 10.02±  0.25 | 6.06±  1.97 | 784±  59 | 56±  32 | 108±  28 | 15.4±  3.2 | 6.8±  2.1 | 20.7±  0.9 |
| US | 10.63±  0.57 | 4.82±  0.95 | 796±  227 | 55±  15 | 154±  39 | 13.7±  2.8 | 6.9±  0.4*^a^* | 9.86±  0.10 | 7.48±  1.91 | 669±  33 | 37±  6 | 122±  31 | 14.2±  2.6 | 6.5±  2.1 | 20.0±  0.9 |
| ZD2767P | 10.65±  0.40 | 6.52±  2.50 | 827±  64 | 58±  8 | 171±  2 | 10.7±  1.6 | 12.4±  2.2 | 9.20±  0.70 | 4.80±  0.41 | 536±  97 | 42±  11 | 127±  40 | 12.2±  3.2 | 7.2±  1.0 | 20.5±  1.4 |
| ZD2767P+US | 9.96±  0.33*^a^* | 8.31±  1.26 | 520±  222 | 44±  5 | 139±  8 | 10.2±  1.6 | 8.5±  2.0*^a^* | 10.19±  0.25 | 3.22±  1.18 | 538±  163 | 54±  23 | 147±  16 | 14.7±  5.9 | 7.6±  0.8 | 20.1±  1.1 |
| CPG2 | 10.57±  0.49 | 5.85±  1.10 | 827±  238 | 57±  14 | 163±  10 | 11.8±  1.3 | 9.2±  0.9 | 8.42±  1.63 | 3.63±  0.34 | 718±  27 | 48±  18 | 122±  9 | 12.5±  4.5 | 8.1±  4.7 | 20.6±  1.5 |
| CPG2+US | 11.28±  0.61 | 4.90±  0.85 | 703±  139 | 56±  14 | 146±  34 | 10.2±  0.9 | 11.1±  2.3 | 10.13±  0.51 | 7.16±  4.29 | 834±  206 | 45±  13 | 131±  10 | 9.4±  0.9 | 7.3±  1.6 | 20.7±  0.9 |
| ZD2767P+CPG2 | 11.19±  0.17 | 5.54±  1.86 | 848±  24 | 66±  7 | 164±  18 | 12.8±  0.3 | 11.5±  1.0 | 10.52±  0.37 | 5.57±  1.97 | 785±  133 | 42±  6 | 137±  38 | 13.0±  0.5 | 5.6±  1.5 | 18.6±  0.5*^a^* |
| ZD2767P+CPG2+US | 10.45±  0.26 | 6.50±  2.41 | 845±  43 | 41±  3 | 144±  22 | 10.2±  0.5 | 7.6±  1.1*^a^* | 10.04±  0.85 | 9.06±  3.92 | 792±  174 | 57±  11 | 155±  47 | 12.8±  0.8 | 7.4±  1.7 | 18.2±  0.3*^a^* |

RBC: red blood cell; WBC: white blood cell; Pt: platelet; Cr: creatinine; BUN: urea nitrogen; ALT: alanine aminotransferase; AST: aspartate aminotransferase; BM: body mass; *^a^*: vs. Ctrl: p < 0.05.
